# Complex disease and phenotype mapping in the domestic dog

**DOI:** 10.1038/ncomms10460

**Published:** 2016-01-22

**Authors:** Jessica J. Hayward, Marta G. Castelhano, Kyle C. Oliveira, Elizabeth Corey, Cheryl Balkman, Tara L. Baxter, Margret L. Casal, Sharon A. Center, Meiying Fang, Susan J. Garrison, Sara E. Kalla, Pavel Korniliev, Michael I. Kotlikoff, N. S. Moise, Laura M. Shannon, Kenneth W. Simpson, Nathan B. Sutter, Rory J. Todhunter, Adam R. Boyko

**Affiliations:** 1 Department of Biomedical Sciences, College of Veterinary Medicine, Cornell University, Ithaca, New York 14853, USA; 2 Department of Clinical Sciences, College of Veterinary Medicine, Cornell University, Ithaca, New York 14853, USA; 3 School of Veterinary Medicine, University of Pennsylvania, Philadelphia, Pennsylvania 19104, USA; 4 Department of Biological Statistics and Computational Biology, Cornell University, Ithaca, New York 14853, USA; 5 College of Animal Science and Technology, China Agricultural University, Beijing 100094, China; 6 Biology Department, La Sierra University, Riverside, California 92505, USA

## Abstract

The domestic dog is becoming an increasingly valuable model species in medical genetics, showing particular promise to advance our understanding of cancer and orthopaedic disease. Here we undertake the largest canine genome-wide association study to date, with a panel of over 4,200 dogs genotyped at 180,000 markers, to accelerate mapping efforts. For complex diseases, we identify loci significantly associated with hip dysplasia, elbow dysplasia, idiopathic epilepsy, lymphoma, mast cell tumour and granulomatous colitis; for morphological traits, we report three novel quantitative trait loci that influence body size and one that influences fur length and shedding. Using simulation studies, we show that modestly larger sample sizes and denser marker sets will be sufficient to identify most moderate- to large-effect complex disease loci. This proposed design will enable efficient mapping of canine complex diseases, most of which have human homologues, using far fewer samples than required in human studies.

## Abstract (web summary)

The domestic dog is an important model organism for our understanding of cancer and other diseases. Here the authors conduct a genome-wide association study across multiple breeds and identify novel loci significantly associated with several complex diseases and morphological traits.

## Graphical abstract

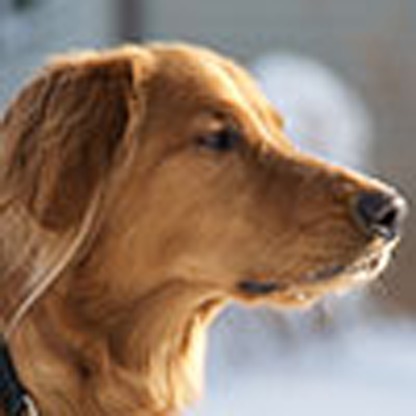

The domestic dog (*Canis lupus familiaris*) is considered to be an excellent animal model for human disease, with over 350 diseases in common—from hip dysplasia to lymphoma—and similar pathways and genes often underlie these shared diseases^1^. Intense artificial selection during the formation of dog breeds has led to diverse morphological and behavioural phenotypes, and, in some cases, considerable differences in disease risk across breeds. Small founder populations and selective breeding have resulted in long regions of linkage disequilibrium (LD) within breeds—over 1 Mb—while across breeds LD is much shorter, more similar to that seen in humans^2^,^3^. This unique breed structure has facilitated the fine mapping of nearly 200 Mendelian traits and disorders, including narcolepsy and cataracts^4^,^5^, using much smaller sample sizes than required in human disease association studies.

The current canine mapping array of 173,000 markers has sufficient power to detect large-effect alleles (≥2-fold risk increase) with 100 cases and 100 controls from a single breed^6^. This mapping approach has identified large-effect risk loci associated with some complex diseases, notably squamous cell carcinoma in the Standard Poodle^7^, atopic dermatitis in the German Shepherd Dog^8^ and canine compulsive disorder in the Doberman Pinscher^9^. Similar to within-breed genome-wide association studies (GWAS) in the dog, there are many studies of complex-disease GWAS in humans using cohorts of related individuals, which are relatively robust (for example, ref. 10). For many canine complex diseases, however, genetic risk is likely determined by numerous loci with small individual effect sizes, and thus larger sample sizes are required for successful association mapping.

Significantly larger sample sizes than those used for canine disease GWAS have been used to discover genes affecting morphological traits, where breed-average phenotypes can be used rather than individual case/control or quantitative data. Large-effect quantitative trait loci (QTLs) have been found for chondrodysplasia^11^, tail curl^12^, ear drop^3^,^12^,^13^, brachycephaly^14^,^15^ and several fur phenotypes^16^. Strong artificial selection for breed-defining morphological phenotypes has likely increased the prevalence of large-effect loci for these traits, and stereotyped the traits within breeds, making breed mapping particularly powerful for identifying genetic associations. For example, a total of 13 loci have been identified that significantly affect weight and/or height in dogs^3^,^11^,^12^,^13^,^17^,^18^,^19^,^20^,^21^,^22^, six of which explain over 80% of the variation in body size in purebred dogs^3^. In comparison, human height is associated with nearly 700 variants, which cumulatively explain only ∼20% of the variation in adult stature^23^.

It is an unanswered question as to what sample sizes and study designs should be employed to improve the power of mapping efforts for complex canine phenotypes^6^,^24^. Current disease-mapping efforts that are focused on single breeds miss much of the genetic variation underlying breed risk that is partitioned across breeds and that may underlie striking differences in disease prevalence across breeds. Conversely, using breed-average phenotypes in multi-breed studies may fail to uncover the genetic variants driving phenotypic heterogeneity within a breed. Larger cohorts of individually phenotyped dogs across multiple breeds are needed to identify the genetic risk factors missed by previous mapping efforts, and provide a better understanding of the genetic architecture underlying complex phenotypes in the dog.

Here we conduct the largest dog genotyping study, with 4,224 samples genotyped on a semicustom 180,000 single-nucleotide polymorphism (SNP) array, in an effort to improve canine mapping for complex traits. Furthermore, we use simulations to determine how different parameters (array marker density, sample size, GWAS design) affect the power to detect causal loci of different effect sizes. Our samples are a heterogeneous mix of purebred dogs representing over 150 breeds, 170 mixed-breed dogs and 350 free-ranging village dogs. The village dogs were sampled from 32 countries worldwide. The samples were gathered by multiple researchers and deposited in the Cornell Veterinary Biobank. Over 65 clinical and morphological phenotypes were considered, although each trait was recorded for only a subset of the cohort. The most common recorded phenotypes were body weight (*N*=2,072), canine hip dysplasia (CHD, *N*=921), elbow dysplasia (ED, *N*=746), cranial cruciate ligament disease (CCLD, *N*=670), mast cell tumour (MCT, *N*=505), lymphoma (*N*=337), portosystemic vascular anomalies (PSVA, *N*=315) and mitral valve degeneration (MVD, *N*=249).

Using GWAS on the 12 most common recorded phenotypes, we find loci associated with several canine complex diseases (including hip dysplasia, granulomatous colitis (GC) and idiopathic epilepsy), as well as novel loci affecting morphological traits, namely body size, fur length and shedding. Furthermore, we use simulations to show that a denser array (SNP spacing of 2 kb) and larger sample sizes (500–1,000 cases and controls) would enable the identification of moderate- to large-effect loci (effect size≥0.5*σ*, that is, a change in the trait at least half as large as the standard deviation of the trait in the population, and minor allele frequency (MAF) ≥5%) underlying a complex phenotype. These results not only validate the dog as a large animal model for the discovery of genes associated with multigenic traits of importance to human medicine, but also demonstrate how to improve canine association-mapping methods for future studies on the genetics underlying complex canine disease.

## Results

### Complex disease phenotypes

Using a univariate linear mixed model implemented in the program GEMMA^25^, we conducted across-breed GWAS for the diseases CHD, ED, CCLD, lymphoma, PSVA, MCT and MVD, and within-breed GWAS for idiopathic epilepsy in Irish Wolfhounds, GC in Boxers and Bulldogs, lymphoma in Golden Retrievers, MCT in Labrador Retrievers, and PSVA in Yorkshire Terriers. GEMMA corrects for population stratification in the data by including a relatedness matrix—calculated from the genotypes—as a random effect. The average inflation factor (*λ*, see Methods) for all GWAS is 1.00 (range of 0.96–1.05), showing that the stratification correction worked well. After Bonferroni correction, we identified seven significant QTLs, and one suggestive QTL, that are associated with complex canine diseases (Table 1).

**Figure 1:**
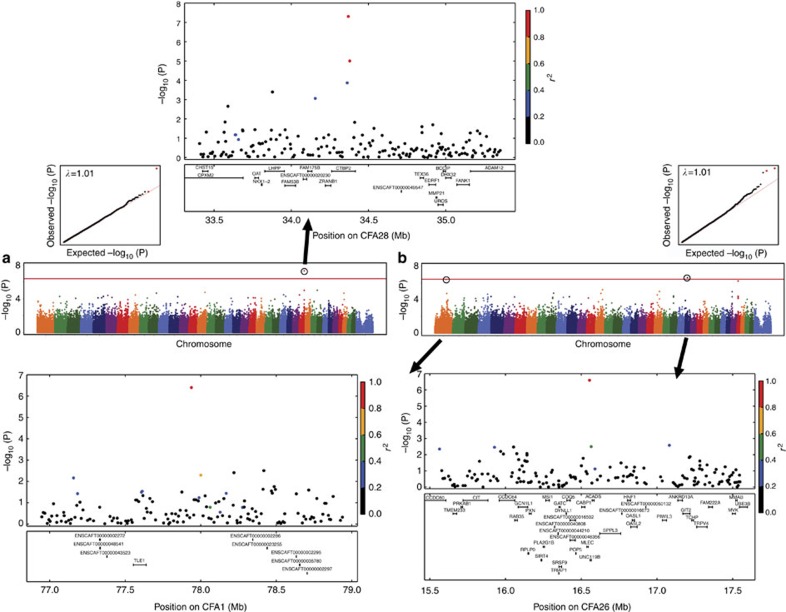
Significant across-breed disease GWAS results. Manhattan and quantile–quantile plots, showing the statistical significance of each marker (−log_10_ scale) as a function of genomic position for (**a**) hip dysplasia (CHD, as measured by Norberg Angle, *n*=921), (**b**) elbow dysplasia (ED, 113 cases and 633 controls). Colours of circles indicate the amount of LD with top-associated marker, ranging from black (*r*^2^=0–0.2) to red (*r*^2^=0.8–1). Red lines on the Manhattan plots are the significance thresholds, at *P*=4 × 10^−7^. Inflation factors (*λ* values) are shown on the quantile–quantile plots.

For CHD, we used a quantitative measure of hip conformation—the Norberg angle (NA)—in 921 dogs across 69 breeds and found an association reaching genome-wide significance on chromosome (CFA) 28 (*P*=4.9 × 10^−8^, *β*=0.07, Wald test) in the gene *CTBP2* (Fig. 1a). CTBP2 acts as a transcriptional corepressor and interacts with the Wnt pathway, which plays an important role in the remodelling of bone^26^. Looking at breeds with the largest sample sizes, we find that the significant CFA28 locus has a major effect (about a 6° additive effect on NA values) in Golden Retrievers and Labrador Retrievers, but not in German Shepherd Dogs (Supplementary Table 1).

Another orthopaedic trait, ED, was studied using 113 cases and 633 controls across 82 breeds, and revealed different associations than CHD, consistent with the low genetic correlation between CHD and ED in dogs^27^. These ED associations are on CFA26 (*P*=2.6 × 10^−7^, *β*=0.18, Wald test) and CFA1 (*P*=4.4 × 10^−7^, *β*=0.17, Wald test), within LD of the genes *POP5* and *TLE1*, respectively (Fig. 1b). POP5 is expressed in cartilage and bone, and is involved in cartilage hair hypoplasia^28^, while TLE1 also interacts with the Wnt pathway and is a specific marker for synovial sarcoma^29^. Variants in the CFA26 locus increase the risk of ED in Golden Retrievers and English Setters, while the CFA1 locus influences ED risk in Labrador Retrievers and German Shepherd Dogs (Supplementary Table 1). We did not find genome-wide significant associations for CCLD, lymphoma, PSVA, MCT or MVD using data across breeds in sample sizes ranging from 249 to 670 (Supplementary Fig 1A–E).

**Figure 2:**
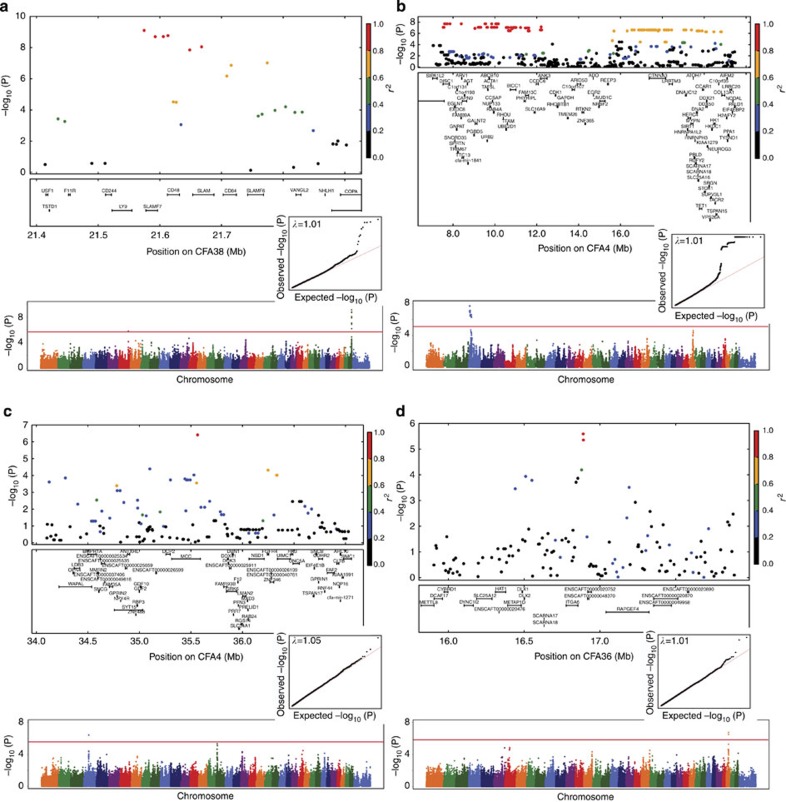
Significant within-breed disease GWAS results. Manhattan and quantile–quantile plots, showing the statistical significance of each marker (−log_10_ scale) as a function of genomic position for (**a**) granulomatous colitis in Boxers and Bulldogs (46 cases, 91 controls), (**b**) idiopathic epilepsy in Irish Wolfhounds (34 cases, 168 controls), (**c**) lymphoma in Golden Retrievers (34 cases, 48 controls), (**d**) MCT in Labrador Retrievers (152 cases, 106 controls). Colours of circles indicate the amount of LD with top-associated marker, ranging from black (*r*^2^=0–0.2) to red (*r*^2^=0.8–1). Red lines on the Manhattan plots are the significance thresholds, calculated by a Bonferroni correction of unlinked markers. Inflation factors (*λ* values) are shown on the quantile–quantile plots.

In contrast to the across-breed GWAS results described above, we found significant associations within breeds for several diseases using very small sample sizes. In Irish Wolfhounds, we found a 13.5-Mb haplotype on CFA4 associated with idiopathic epilepsy in 34 cases and 168 controls (*P*=2.0 × 10^−8^, *β*=0.16, Wald test; Fig. 2b). This haplotype—at 38% frequency in cases and 11% in controls—contains several candidate genes for the disorder, including *ANK3*, *EGR2* and *SLC16A9*. In Boxers, we identified a 400-kb region on CFA38 associated with *Escherichia coli*-associated GC^30^ in 40 cases and 74 controls (*P*=1.6 × 10^−8^, *β*=0.34, Wald test; Supplementary Fig. 2). As French Bulldogs and American Bulldogs are related breeds with a similar presentation of GC^31^, we included an additional 6 bulldog cases and 17 bulldog controls, further strengthening the genetic association at this locus (*P*=8.1 × 10^−10^, *β*=0.33, Wald test; Fig. 2a). This region is also associated with inflammatory bowel disease in humans^32^ and includes members of the *SLAM* family, which have been shown to be important in mounting responses to bacterial infection in mice^33^. In Golden Retrievers, we found a QTL on CFA4 associated with lymphoma in 34 cases and 48 controls (*P*=4.0 × 10^−7^, *β*=0.49, Wald test; Fig. 2c) containing several candidate genes, including *MCC*, *MXD3* and *FGFR4*. Finally, GWAS of MCT in Labrador Retrievers (152 cases, 106 controls) revealed a significant QTL on CFA36 (*P*=1.7 × 10^−7^, *β*=0.36, Wald test; Fig. 2d), within 30 kb of the candidate gene *ITGA6*.

For other complex diseases, we did not have sufficient cases and/or controls within single breeds to find significant associations, although we saw a suggestive association on CFA32 for PSVA in Yorkshire Terriers, which warrants further investigation (*P*=1.2 × 10^−6^, *β*=0.48, Wald test; Supplementary Fig. 1F). We looked at the frequencies of these within-breed associations for lymphoma, MCT and PSVA in other main dog breeds included in our data set, and found that these loci only affect disease risk in Golden Retrievers, Labrador Retrievers and Yorkshire Terriers, respectively (Supplementary Table 1).

### Breed-level morphological phenotypes

**Figure 3:**
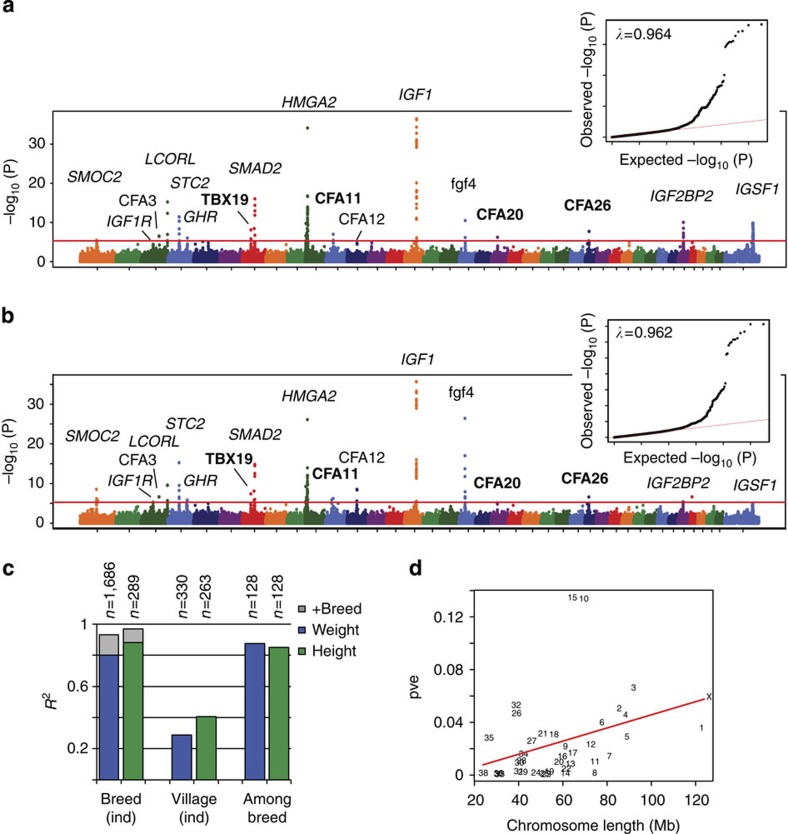
Body size association results. Manhattan and quantile–quantile plots of (**a**) breed-average male weight^0.38^ (*n*=1,873) and (**b**) breed-average male height (*n*=1,873), showing the 17 significant loci, four of which are novel (shown in bold). Red lines on the Manhattan plots are the significance thresholds, at *P*=5 × 10^−6^ (FDR of <0.5% and <0.75% for weight and height, respectively). Inflation factors (*λ* values) are shown on the quantile–quantile plots. (**c**) Proportion of variance explained (*R*^2^) by the 17 size loci in a linear model for weight (blue bars) and height (green bars), with sex and inbreeding corrections. Shown are the results for individual breed dogs (with breed included in the model shown in grey), individual village dogs and among breeds. (**d**) Proportion of variance explained (pve) by SNPs on each chromosome for individual weight (*n*=2,072) by the length of the chromosome. Points are plotted as chromosome numbers.

To investigate the genetic basis for complex morphological phenotypes that differ across breeds, we used breed-level measures of body size and fur characteristics. Specifically, we ran quantitative GWAS using 1,873 dogs from 158 breeds, with each dog assigned its breed phenotype (male breed average weight (transformed by x^0.38^), height (untransformed), fur length on a scale from 1 to 5, fur shedding on a scale from 0 to 1). We found 17 significant associations with male breed-average weight and/or height at a false discovery rate (FDR)<0.5% and <0.75%, respectively (Fig 3a,b). In addition to known body size loci including *IGF1*, fgf4, *HMGA2* and *IGF1R*^3^,^11^,^12^,^13^,^17^,^18^,^20^, we identified four novel loci on CFA7 (30,243,851), CFA11 (26,929,946), CFA20 (21,479,863) and CFA26 (13,224,865) (Supplementary Fig. 3). The CFA26 novel locus was no longer significant after applying stepwise analysis where associated markers were included as covariates to reduce spurious allelic association^34^; therefore we can conclude that three novel loci have been identified (Supplementary Table 2). The CFA7 locus is approximately 10 kb upstream of the gene *TBX19* (Supplementary Fig. 3A), which is expressed in the pituitary gland^35^. The CFA20 locus is about 300 kb downstream of *MITF* (Supplementary Fig. 3C), which is known to cause white spotting in dogs^36^ but has also been associated with body weight in quail and mice^37^,^38^.

With the large number of individuals and breeds in our data set, we could further refine the association intervals at other body size loci (Supplementary Fig. 4), including narrowing a 3-Mb association interval on CFAX (101–104 Mb) down to a 700-kb region containing three genes: *ARHGAP36*, *IGSF1* and *OR5AK2* (Supplementary Fig. 4B). *IGSF1* is a particularly promising candidate gene as it is linked to changes in growth and size in humans^39^.

**Figure 4:**
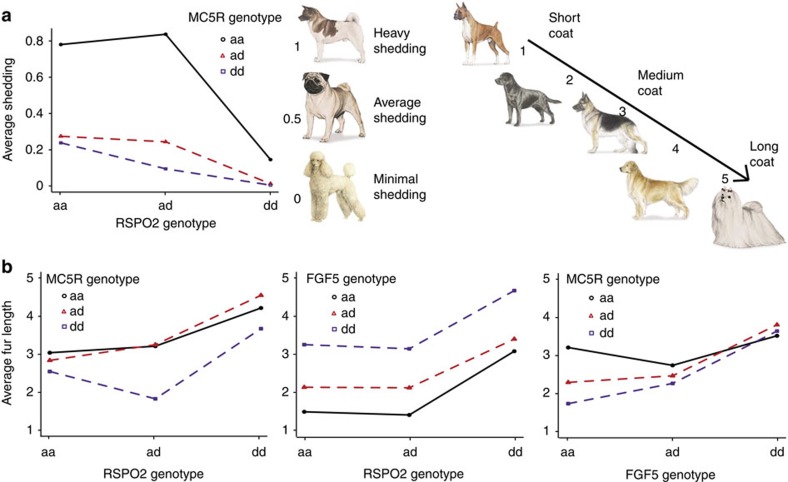
Epistasis plots for fur phenotypes. (**a**) Breed average shedding (on a scale from 0=minimal to 1=heavy), showing the interaction between *RSPO2* and *MC5R* alleles, (**b**) breed fur length (on a scale from 1=short to 5=long), showing the interaction between *MC5R* and *RSPO2* alleles, *FGF5* and *RSPO2* alleles, and *MC5R* and *FGF5* alleles. a=ancestral allele, d=derived allele. Breed images are used with permission from the American Kennel Club (AKC).

For the breed-mapped phenotype of fur length, we used a 1 (short hair) to 5 (long hair) phenotypic scale and identified a novel locus on CFA1 (*P*=2.2 × 10^−12^, *β*=0.16, Wald test) in addition to the known fur genes *FGF5* (located on CFA32, *P*=3.1 × 10^−44^, *β*=0.31, Wald test) and *RSPO2* (located on CFA13, *P*=2.0 × 10^−28^, *β*=0.26, Wald test)^16^ (Supplementary Fig. 5A). This most associated variant at the novel CFA1 locus (at 24,430,748) is a missense mutation in *MC5R* that changes the ancestral alanine to the boxer reference threonine (A237T), and was included as a custom marker on the CanineHD array because it was observed to be segregating in village dog whole-genome sequences^40^. The *MC5R* protein sequence is evolutionarily conserved across mammals, and we find evidence that the A237T mutation causes a conformational change in the tertiary structure of the protein, with a change in binding sites (Supplementary Fig. 6) that is ‘probably damaging' (Polyphen-2 HumDiv=0.992). *MC5R* is expressed in human sebaceous glands and is involved in the production of sebum in mice, affecting water repellency and thermo-regulation^41^,^42^. While functional studies are needed to determine whether this variant is indeed the causal mutation at this QTL, the identification of a third coat length locus improves our understanding of fur-type genetics in the dog and hypothesizes a relationship between sebum production and fur type in some breeds. Longer-haired breeds (for example, Maltese, Old English Sheepdog) are homozygous for the derived *FGF5* allele, with the presence of the ancestral *RSPO2* allele distinguishing medium–long from long hair^16^ (Fig. 4b). Shorter-haired breeds (for example, Bull Terrier, Greyhound) have the ancestral *FGF5* allele and the derived *MC5R* allele, while medium-haired breeds (for example, Akita, Pembroke Welsh Corgi) have the ancestral *MC5R* allele (Fig. 4b).

*MC5R* and *RSPO2* were also significantly associated with fur shedding (*P*=5.9 × 10^−17^, *β*=0.057, Wald test, and *P*=9.8 × 10^−12^, *β*=0.047, Wald test, respectively; Supplementary Fig. 5B). Minimal-shedding breeds (for example, Poodle, Bichon Frisé) are homozygous for the derived *RSPO2* allele (Fig. 4a). Heavy-shedding breeds (for example, Akita, Alaskan Malamute) are homozygous for the ancestral *MC5R* allele in the presence of the ancestral *RSPO2* allele, while medium-shedding breeds (for example, Cocker Spaniel, Pug) have the derived *MC5R* allele in the presence of the ancestral *RSPO2* allele (Fig. 4a).

### Individual-level weights

We compared the power of using breed-average weights versus individual body weights for 2,072 dogs, including 330 village dogs, and also compared the genetic architecture of body size between purebred lines and natural dog populations. Using individual weights on 2,072 dogs results in a loss of GWAS power compared with using breed-level phenotypes from 1,873 dogs (as shown by the *P*-values in Supplementary Table 2). Nearly all 17 breed-level size QTLs showed reduced significance in the individual-level association and no new associations became evident in GWAS using individual data from purebred or village dogs (Supplementary Fig 5C,D, Supplementary Table 2).

Using an additive linear model where we corrected for both inbreeding and sex of the dog (see Methods), we confirm that dog body size has a simple underlying genetic architecture^3^,^13^, with the identified 17 QTLs explaining 80–88% of the variation of weight and height in individual purebred dogs (Fig. 3c; Supplementary Table 3). Including breed in the model increases the fit to over 90%, suggesting the presence of rare or small-effect QTLs not identified in our GWAS that also contribute to breed differences (Fig. 3c). In village dogs, the linear model only explains 30–40% of body size variation, highlighting that the genetic architecture for dog body size has been greatly impacted by disruptive selection for size in purebred dog lineages. In contrast to the genetic architecture of human morphological traits, such as height^43^, we see that the chromosomes with large-effect loci (such as 3, 10 and 15) explain much of the variation in canine body weight (Fig. 3d). However, consistent with human population studies^44^, we find that, within a breed, inbred dogs tend to be smaller than outbred dogs, a likely consequence of deleterious recessive variants having negative effects on growth when homozygous. For example, using the results of our linear model, in a breed that has an average male weight of 20 kg, an individual with an inbreeding coefficient elevated 10% would be expected to be 1.2 kg smaller than average.

### Simulations

**Figure 5:**
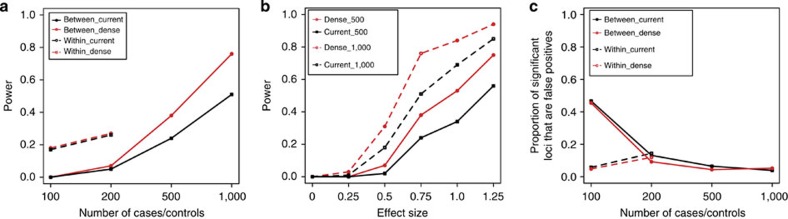
Simulation GWAS results. (**a**) Between-breed and within-breed GWAS designs using a dense (1 SNP every 2 kb) array (red) and the current (1 SNP every 13 kb) array (black) with different numbers of cases and controls. Shown is the power to detect causal loci with effect size of 0.75*σ*. (**b**) Power to detect loci of different effect sizes using a between-breed GWAS design and a dense array (red) and current array (black) with 500 cases/controls and 1,000 cases/controls. (**c**) Proportion of significant loci that are false positives using a dense (red) and the current (black) array with between-breed and within-breed GWAS designs and different numbers of cases and controls.

Through a simulation study of a complex trait with five causal loci (all with a MAF of 5–10%), 50% heritability and 20% liability, we find that the power to detect causal loci increases with an increase in number of cases and controls (Fig. 5a). For a given sample size, a within-breed GWAS design has higher power at lower sample sizes than an across-breed design (for example, the percentage of causal loci with effect size of 0.75*σ* that are identified using 200 cases and controls is 26–27% versus 5–7% for a within-breed versus across-breed design). However, using across-breed GWAS designs, we can more easily increase sample sizes up to at least 500 cases and controls, where the power to identify the same loci increases to 24% using the current CanineHD platform (Fig. 5a). Furthermore, using an array with a marker density of 1 SNP every 2 kb, the power to identify the same loci increases to 38%, as the denser marker spacing enables accurate tagging of causal loci (Fig. 5a). Increasing the sample size to 1,000 cases and 1,000 controls yields even greater power to detect moderate-effect loci (Fig. 5b).

We examined the false discovery rate (FDR), calculated as the proportion of significant loci that are false positives, in our across-breed and within-breed simulation GWAS. For across-breed GWAS designs including more than 200 cases and controls, FDR plateaus around 5% (Fig. 5c). For the within-breed design, FDR increases with an increase in sample size, as the number of false positives increases at a rate higher than that of true positives. Importantly, FDR does not significantly differ between the dense and current arrays, suggesting that our thresholds of *P*=1 × 10^−7^ and *P*=5 × 10^−7^ respectively, are appropriate.

Using an across-breed GWAS design, we see an increase in power with an increase in sample size, an increase in effect size, and increased SNP density, while the latter does not apply to a within-breed design (Supplementary Fig. 7A), since LD within a breed does not break down as rapidly as across breeds. Furthermore, different across-breed GWAS designs (balanced, semi-balanced, unbalanced, random; see methods) do not significantly affect the power to detect causal loci, showing that a random design can be just as powerful as a balanced design (Supplementary Fig. 7B).

## Discussion

In this study, we generate the largest canine genotyping data set so far, with 4,224 dogs genotyped at 180,000 markers. We expand the number of complex disease loci and morphological QTLs known in the dog and, importantly, demonstrate the efficacy of genetic mapping in heterogeneous populations of dogs. We identify significant associations using an across-breed mapping approach for CHD and ED, and a further four significant associations within breeds for lymphoma, GC, idiopathic epilepsy and MCT. The colitis association is particularly exciting because the region has also been identified in an inflammatory bowel disease (IBD) GWAS study in humans^32^, further supporting the usefulness of the domestic dog as a natural animal model for human diseases. Our findings differ from previously published studies of canine associations with lymphoma^45^, orthopaedic diseases^46^,^47^,^48^ and idiopathic epilepsy^49^, although we use different breeds, different phenotypic criteria and, in most cases, a larger number of samples. Discrepancies between different studies is not surprising given the differences in study designs and sampling cohorts, and highlight the need for follow-up validation studies.

Despite large sample sizes, our scans failed to detect significant associations in across-breed GWAS for several complex diseases (CCLD, lymphoma, PSVA, MCT and MVD). While a larger genotyped cohort may be needed for some diseases due to genetic architecture and environmental effects, our simulations show that increased marker density is also needed to improve mapping power across breeds. A decrease in mean marker spacing from 13 kb (the current CanineHD array spacing) to 2 kb results in an increase in power for moderate-effect loci. Based on human complex disease studies^50^, we believe that much of the genetic basis for most canine complex diseases will be shared across breeds, but that small sample sizes and variable or poor tagging of causal variants across breeds has been the cause of the inconsistent and insignificant associations for these diseases in canines thus far. To the extent that causal variants segregating at different allele frequencies in different breeds drive differential disease risk across breeds, association studies using individuals from multiple breeds will be a powerful mapping strategy for complex canine phenotypes, but, at this point, it is unclear to what extent shared versus breed-specific variants drive the genetic risk for complex canine diseases.

In contrast to disease traits, the mapping results for the morphological phenotypes of fur length and body size reveal highly significant associations that are consistent with earlier studies^3^,^11^,^13^,^16^,^19^. The increased sample size and marker density of this study enabled identification of at least three novel body size QTLs, and a novel association of *MC5R* with fur length and shedding. Because these loci have undergone selective sweeps due to strong artificial selection for fur phenotype and size, relatively large haplotype blocks and high LD surrounding the causal variants facilitate genetic mapping by improving the likelihood that array markers accurately tag each causal variant. Nevertheless, improving marker density even at positively selected loci will improve power. If the *MC5R* A237T mutation had not been identified in resequencing data sets and included on this array, the association between CFA1 and fur length would not have reached Bonferroni significance and may have been missed. As resequencing data sets do not show other coding variants in this region in high LD with the A237T mutation^40^, we believe this missense mutation may be the causal variant for the phenotype, although functional studies are needed to establish this.

Unlike previous canine body size studies, we used individual phenotypes as well as breed averages to detect genetic associations. Using breed-average size data resulted in a clear increase in GWAS power for detecting body size associations, with *P*-values up to several orders of magnitude higher for most of the QTLs, compared to using the individual-size data. This difference is likely due to the extreme variation seen in dog body size across the different breeds and the greater role of environmental effects, such as diet, on individual weights compared to breed averages. For disease traits, multi-breed mapping using individual data is needed in order to capture both across-breed and within-breed genetic variation in association studies. Importantly, a balanced GWAS design is not necessary; using dogs that are randomly chosen across different breeds seems to be just as powerful as using a balanced number of cases and controls from each breed.

Our modelling indicates that future studies consisting of 500–1,000 cases and 500–1,000 controls from numerous purebred and mixed-breed populations, with denser marker spacing through denser genotyping arrays and/or imputation panels, will substantially increase the number of loci known to affect canine complex diseases, many of which are homologous to human disorders. This finding suggests that studies much smaller than those currently used in human GWAS designs will yield important genetic associations, making dogs an attractive model for studying and mapping complex phenotypes.

## Methods

### Sample collection

Blood samples were collected in accordance with Cornell University animal care and use guidelines (IACUC #2005-0151 and #2011-0061). Genomic DNA was extracted using a standard salt precipitation from EDTA blood samples and stored in the Cornell Veterinary Biobank. Phenotypes were recorded at the time of blood collection, but disease phenotypes continued to be updated during subsequent veterinary examinations.

### Genotyping

Genotyping was done using the Illumina 173k CanineHD array^12^, with the addition of 12,143 custom markers (see PLINK files deposited in Dryad). These custom markers were SNPs that were identified from whole-genome sequence data^40^, variants listed in Online Mendelian Inheritance in Animals (OMIA, http://omia.angis.org.au/home/), and markers that were designed to cover gaps in the existing array (including mitochondrial DNA and the Y chromosome). In total, 4,224 samples were genotyped (44 plates) at 185,805 markers at the Cornell University core facility. All positions are listed in canFam3.1.

PLINK data sets were generated in GenomeStudio using the PLINK report plugin. Genotypes were called using a GenCall threshold of 0.15 using cluster positions that were computed for the first 30 plates. In PLINK v1.07 (ref. 51), SNPs with a genotyping rate below 95% were removed. Duplicate samples were merged and discordant SNPs between the duplicates were identified and removed. SNPs with a MAF over 2% were tested for unexpected deviations from Hardy–Weinberg equilibrium. Specifically, SNPs with heterozygosity ratios (observed versus expected number of heterozygotes under Hardy–Weinberg equilibrium) below 0.25 or above 1.0 were identified and removed. Furthermore, all Y chromosome and mitochondrial DNA SNPs with any heterozygous genotype calls were removed. In total, 180,117 SNPs remained after filtering, with an overall call rate of >99.8%. The concordance rate between 44 technical replicates was 99.99%.

Samples with >10% missing genotypes or with recorded sex not matching genotypic sex were excluded from further analysis. Genotypic sex was computed by calculating (1) the proportion of missing Y chromosome genotypes (<50% in males, >50% in females) and (2) the homozygosity across non-PAR X chromosome markers using the PLINK --check-sex option (generally <60% in females, >60% in males). In this manner, XXY samples were not misidentified as females and females with highly inbred X chromosomes were not misidentified as males. To check the recorded breed of our samples, we used the PLINK --genome option to check that each individual is most closely related to other individuals of the same breed, and we also ran a principal component analysis (PCA) on each breed using the program EIGENSTRAT in the EIGENSOFT v5.0.1 package^52^ to identify any outliers. Dogs with recorded breed not matching the genotypic breed were excluded from further analysis.

Phasing was performed on the data set to eliminate missing genotypes through imputation, allow for haplotype-based association, and to facilitate merging data from the custom chip to published and unpublished CanineHD data sets for meta-analysis^12^. Briefly, phasing was done for all autosomal and X chromosome markers with MAF>0.01 with the first 30 plates. Additional custom plates or CanineHD datasets were pre-phased using SHAPEIT^53^, and then phased with IMPUTE2 (ref. 54) using the data set from the first 30 plates as the imputation reference panel.

### GWAS

All GWAS were run using a linear-mixed model in GEMMA v.0.94 (ref. 25). Unless otherwise stated, no covariates were included in the GWAS. The genotype data were pruned to include only individuals with phenotypes for the trait of interest, and only SNPs with MAF >0.05 in these individuals were included in the calculation of the kinship matrix. This kinship matrix is included as a random effect in the association and we use the Wald test to determine *P*-values. To ensure sufficient correction for population stratification, we calculated inflation factors (*λ*) in R (ref. 55) for each association. Inflation factors compare the median test statistics from the data and the expected null distribution, with a value of 1.0 representing no inflation.

A significance threshold of *P*=4 × 10^−7^ (Bonferroni cutoff of *α*=0.05) was used for across-breed GWAS; for within-breed GWAS, the significance threshold was based on the Bonferroni cutoff of SNPs that were not in complete or near-complete LD as calculated using the PLINK command --indep 100 10 10. For within-breed GWAS, PCA was first run to identify and remove outlier individuals.

Case/control or quantitative GWAS was performed on subsets of dogs using individual clinical phenotype data, as described below. Numbers of dogs in each breed included in each GWAS are listed in Supplementary Data 1. Manhattan and quantile–quantile plots were constructed in R. LD plots were generated using Matplotlib library^56^ in IPython notebook^57^.

*Orthopaedic traits*: Dogs used in GWAS of orthopaedic traits were selected from the following four sources: Cornell University Hospital for Animals, The Baker Institute for Animal Health at Cornell University, Guiding Eyes for the Blind in Yorktown Heights, NY, and the Orthopedic Foundation for Animals.

1. *Hip dysplasia*: CHD is the most common orthopaedic trait in medium- and large-breed dogs, with incidences ranging from less than 10% to over 70% across purebred dogs. The diagnosis of dysplasia was initially made on orthopaedic exam, but confirmed radiographically. The measure of CHD used was the NA, which is measured on a ventrodorsal extended-hip radiograph, and ranges from <20° (worst) to 120°. We ran a quantitative GWAS on the average NA score for the two hips of each dog across 69 breeds (and including 121 mixed-breed dogs) (*N*=921), where all dogs were over 5 months of age and the lowest NA was set to 75 to reduce outlier effects. Adjusting the NA score for the age of the dog at diagnosis did not affect the results.

2. *Elbow dysplasia*: ED is a group of disorders that affect the articular surfaces of the elbow or elbow congruency. The three most common forms are fragmented medial coronoid process, osteochondritis dissecans and ununited anconeal process. Radiographs are useful for diagnosing ununited anconeal process and sometimes osteochondritis dissecans, but the best diagnostic method for the fragmented medial coronoid process is computed tomography. The Orthopedic Foundation for Animals uses a flexed lateral radiograph taken at ≥2 years of age, and radiologists look for signs of secondary osteoarthritis, which always occur with this condition. ED was diagnosed radiographically and, in a small number of cases, confirmed by computed tomography or arthroscopy. Control dogs were over 2 years of age and had a normal flexed lateral radiographic examination. GWAS was performed on 113 cases and 633 controls in 82 breeds (and including 20 mixed-breed dogs). In total, 476 of the ED dogs were also included in the hip dysplasia GWAS.

3. *Cranial cruciate ligament disease*: CCLD is the most debilitating orthopaedic trait affecting the hind limb of dogs. Ruptures were diagnosed by palpation followed by stifle radiographs or by arthroscopy/arthrotomy during surgical correction. Control dogs were over 8 years of age and were subjected to careful orthopaedic examination, specifically feeling for stability on stifle palpation (no cranial drawer or cranial thrust) and/or stifle radiography. GWAS was done using 271 cases and 399 controls across 68 breeds (and including 53 mixed-breed dogs).

*Mitral valve degeneration*. MVD accounts for nearly three-quarters of all cardiovascular diseases in dogs, with small-breed dogs (<9 kg) more commonly affected^58^. Clinical presentation includes a systolic murmur heard loudest on the left side of the thorax, coughing and exercise intolerance. Clinical signs often progress to congestive heart failure. MVD diagnosis was confirmed with the presence of valve leaflet thickening and colour-flow Doppler evidence of mitral valve regurgitation during echocardiographic examination. Control dogs were over 10 years of age and had no evidence of cardiac disease at echocardiographic examination (no thickening or regurgitation). GWAS was performed using 154 cases and 95 controls from 32 small breeds.

*Mast cell tumour*: MCTs are the most common skin tumour in the dog, with a reported annual incidence of 126 cases per 100,000 dogs^59^. The average age at presentation is 8 years, but MCTs are occasionally found in younger dogs and there is no apparent sex predilection. Histological grading is commonly used as a prognostic tool for canine cutaneous MCTs. The most widely used grading system is that by Patnaik *et al*.^60^, which identifies three histological grades. For our analysis, all three tumour grades were represented and no significant difference in results was seen when each affected grade was analysed independently.

Phenotypic criteria included cytologic and/or histologic confirmation of the tumour at excisional biopsy and histologic grading. Control dogs (more than 8 years of age at presentation and not diagnosed with any other type of cancer) had all skin masses mapped and fine-needle aspirated by board-certified oncologists, and the cytology of all masses was reviewed to confirm the absence of mast cells. We performed a GWAS for MCT using 359 cases and 146 controls across 41 breeds, and another GWAS for Labrador Retrievers only, which included 152 cases and 106 controls after removal of PCA-identified outliers.

*Lymphoma*: Lymphoma is the most common haematopoietic tumour of dogs, with a reported annual incidence of 79–103 cases per 100,000 dogs, with annual incidence rates in some breeds above 200 per 100,000 dogs^61^. Lymphoma diagnosis was based on histologic or cytologic confirmation of the tumour, and immunophenotyping (done by immunohistochemistry, flow cytometry or PCR for antigen receptor rearrangements) was used to determine the cell type of the tumour (B or T cell). Our GWAS included 199 cases and 138 controls across 59 breeds (and 2 mixed-breed dogs). Of the 199 cases, 94 are B-cell, 44 are T-cell, 3 are biphenotypicals and the remaining cases were not immunophenotyped. Control dogs were over 8 years of age and had a complete physical examination performed by a board-certified oncologist and all palpable peripheral lymph nodes evaluated for enlargement. We also performed a GWAS using Golden Retrievers only, with 34 multicentric lymphoma cases and 48 controls. In this within-breed GWAS, nine golden retrievers were excluded for their lymphoma type (four epitheliotropic and five gastrointestinal lymphomas).

*GC in boxers and bulldogs*: GC is a severe inflammatory bowel disease (IBD), typically diagnosed in Boxers and Bulldogs younger than 4 years of age. It is characterized by periodic acid-Schiff-positive macrophages and mucosally invasive *E. coli*^30^,^31^. Affected dogs typically present with haemorrhagic diarrhoea, often progressing to chronic weight loss, anaemia, hypoalbuminaemia and debilitation. In our GWAS, we used a panel of 114 Boxers, 22 French Bulldogs and 1 American Bulldog, consisting of 46 cases less than 4 years of age, and 91 controls over 7 years of age. Affected dogs were GC- and *E. coli*-positive, while unaffected dogs were non-GC with no invasive bacteria. Forty individuals were previously genotyped on the CanineHD array and merged with the current data set.

*Portosystemic vascular anomaly*: PSVA is characterized by a severe malformation of the portal vein that carries splanchnic blood to the liver, and mainly afflicts small-breed dogs. Dogs with PSVA fail to detoxify substances in blood, resulting in high serum bile acid concentrations, seizures, lethargy and vomiting. Total serum bile acid (TSBA) values in young dogs (<4 years) were used to directly indicate phenotype status, whereas TSBA values in older dogs (>5 years) were interpreted in the light of that dog's health status (that is, medical history and current medications) to rule out misinterpretation of acquired liver disorders as congenital vascular malformations. Control dogs had TSBA concentrations <25 μmol l^−1^, while cases had TSBA concentrations >25 μmol l^−1^. We conducted a PSVA GWAS using a balanced case/control design of eight small-sized breeds (Cairn Terrier, Havanese, Maltese, Miniature Schnauzer, Norfolk Terrier, Papillon, Tibetan Spaniel, Yorkshire Terrier) and small-breed mixes, for a total of 160 cases and 155 controls. Our GWAS on Yorkshire Terriers included 57 cases and 101 controls, after removal of five dogs from the data set that were PCA outliers.

*Idiopathic epilepsy in Irish Wolfhounds*: Idiopathic epilepsy was diagnosed by exclusion of other causes for seizures in all of the affected dogs. In a previous study of 796 Irish Wolfhounds, 146 dogs were diagnosed as having idiopathic epilepsy^62^. Males were more commonly affected than females, and 73% of the dogs experienced their first seizure by the age of 3 years. We performed a GWAS of idiopathic epilepsy in Irish Wolfhounds using 34 cases and 168 controls. Idiopathic epilepsy was confirmed by the clinical presence of seizures and affected animals had routine blood work, urinalysis, and a full neurologic examination. Select cases had metabolic screening, magnetic resonance imaging, and an electroencephalogram. Control dogs were 6 years of age or older, with no history of seizures.

*Breed mapping*: Using a pruned data set of 1,873 unrelated individual dogs (maximum of 25 dogs per breed), from 158 breeds, we performed breed-average GWAS on several different morphological phenotypes: shedding, fur length, body weight and height at withers. Ancestral alleles for specific loci were determined by looking at the genotypes of a culpeo fox and wolf samples^12^ that were also genotyped on the CanineHD array.

*Body size*: Average male body weights were assigned to breeds based on American Kennel Club standards, CanMap^3^, the Royal Canin dog encyclopedia^63^ and North Carolina Responsible Animal Owners Alliance website (www.ncraoa.com) and compared to the averages of individual measurements from the Canine Behavioral Assessment and Research Questionnaire^64^ and unpublished Cornell databases. Average male height at withers values for breeds were collected from American Kennel Club standards and Frynta *et al*.^65^.

Weight was run as a quantitative trait using the breed-average male weight^0.38^, as determined by Box–Cox transformation. Significance cutoff was set to *P*=5 × 10^−6^ (FDR of <0.5%, <0.75% and <0.95% for breed-average weight, height and individual weight, respectively). The raw breed-average male heights were used in the GWAS, also run as a quantitative trait. Breed-average values used in the GWAS are listed in Supplementary Data 2. A GWAS was also run for individual weights (*N*=2,072) measured in dogs over 1 year of age. Sex-corrected weights were computed by increasing female weights by 19% to account for the sexual dimorphism that we observed.

Using the function lm in R, we ran a linear additive model of corrected weight^0.38^ and corrected height based on the 17 QTLs identified from the GWAS study to identify the additive effect of each locus. Observed weights and heights were corrected by first increasing female weights by 19% and female heights by 8% (based on the sexual dimorphism observed for these traits in our data). After applying a 0.38-power transformation to the body weights, inbreeding correction based on the inbreeding coefficient, *F*, estimated by Germline v1.5.1 (ref. 66) was done by adding *F* × 0.604 and *F* × 3.025 to the transformed weights and heights, respectively. These inbreeding depression parameters were determined from fitting a linear model using individual sex-corrected weights (or heights) for all purebred dogs and setting *F* and breed as independent variables. These corrected weights and heights were used in linear models for each of the data sets: breed dogs (excluding breeds with less than three individuals) and village dogs.

To determine the effect of the QTLs within breeds, we ran the original linear model, but with an independent variable, breed, as well as the 17 QTLs. To determine the effect of the QTLs among breeds, we ran the same linear model using (male) breed averages instead of individual heights and weights. For all linear models using individual phenotypes, QTL variables were encoded as 0/1/2 based on the derived allele count for each individual; for the linear models using breed averages, derived allele frequencies within each breed were used. No inbreeding correction was made for models using breed average phenotypes.

For individual body weight in dogs (*N*=2,072), we used GCTA^67^,^68^ to determine the proportion of the phenotypic variance explained, partitioned into chromosomes. We used restricted maximum likelihood (REML) analysis with an expectation–maximization (EM) algorithm, and full X chromosome dosage compensation, as has been used in human height and BMI studies^43^.

*Fur length*: Phenotype information was collected from Cadieu *et al*.^16^, but we had extra intermediate categories (scale of 1 to 5), determined by visual inspection of breeds. Hairless breeds (for example, Xoloitzcuintli) were excluded from the analysis. Fur length was run as a quantitative trait, with breeds categorized as short (*N*=431, 33 breeds), medium–short (*N*=159, 12 breeds), medium (*N*=279, 29 breeds), medium–long (*N*=548, 42 breeds) and long (*N*=368, 31 breeds) (Supplementary Data 2).

PolyPhen-2 (ref. 69) was used to predict the consequence of the postulated causal mutations in humans, using the HumDiv- and HumVar-trained models. MC5R protein structural and functional features were predicted using PredictProtein (http://www.predictprotein.org) and the 3D structure was modelled in Swiss Model (http://swissmodel.expasy.org) using 3EML as the template.

*Shedding*: Phenotype information was collected from eight different websites: akc.org, dog-breeds.findthebest.com, www.1800petmeds.com, dogtime.com, www.dogbreedinfo.com, www.vetstreet.com, www.petstew.com and www.yourpurebredpuppy.com. Seasonal shedders (for example, Golden Retrievers) and hairless breeds (for example, Xoloitzcuintli) were excluded from the analysis. Shedding was mapped by assigning dog breeds into the categories of minimal (*N*=974 individuals, 81 breeds), average (*N*=359, 28 breeds) and heavy (*N*=205, 15 breeds) shedders, assigned a value of 0, 0.5 and 1, respectively, to be used in quantitative GWAS (Supplementary Data 2).

### Population genetics simulations

To simulate canine complex genetic disease, we used the program GENOME^70^ to generate 30 purebred populations with *N*_e_=250–1,500 depending on population, founded from a larger ‘village dog' population (*N*_e_=30,000) 200 generations ago, which itself was founded from a ‘wolf' population with *N*_e_=15,000, 4,000 generations earlier after a 150-generation bottleneck (*N*_e_=600). We simulated 38 chromosomes (50 Mb per chromosome) with a mutation rate of 1e^−8^ and recombination rate of 1e^−5^. To ensure we could sample 500 diploid individuals (1,000 haplotypes) per population, we simulated larger population sizes (*N*_e_=2,000) for the purebred populations for the final four generations (see Supplementary Methods for the full commands). These parameters roughly correspond to what is known about dog population history and result in LD decay and *F*_ST_ patterns qualitatively consistent with our data.

To ascertain variants for genotyping, we randomly selected two individuals (each from a different purebred population), and selected segregating variants from those individuals either uniformly or based on the distribution of inter-SNP distances for the CanineHD array. For selecting uniform variants, we selected variants in order by selecting the subsequent ascertained variant in the simulation closest to 2 kb (or 10 kb) downstream. For selecting variants with CanineHD-like spacing, we permuted the inter-marker distances between consecutive markers on the CanineHD array, and then selected the subsequent simulated ascertained variants closest to this distance from the previous included variant.

To compare population genetic parameters between our simulation and the purebred populations in our study, we randomly selected 25 individuals from each simulated purebred population and compared these to the 31 breeds in the pruned data set containing exactly 25 individuals. We compared the CanineHD-like ascertained variants from our simulation to our array data, and found that the simulated *F*_ST_ values and rates of LD substantially overlapped for the simulated and observed populations. Across all populations, weighted *F*_ST_ was 0.24 in the simulated populations versus 0.226 in the observed data (pairwise *F*_ST_ range was 0.12–0.51 versus 0.08–0.30). LD decay was somewhat faster within the simulated populations, with mean *r*^2^ being 0.247 (range 0.14–0.47) versus 0.235 (range 0.126–0.411) for autosomal markers 95–112.5 kb apart within simulated and observed populations, respectively. Thus, the simulation captures key aspects of dog population structure that influence mapping, but may somewhat underestimate the power to detect QTLs in real dog populations if LD is greater in those populations.

For simulating complex diseases, we used the GCTA --simu-qt option with five randomly selected causal variants (--simu-causal-loci) chosen from a list of all variants at 5–10% cumulative MAF across the 30 populations. Effect sizes were randomly assigned to the causal loci such that each iteration included effect sizes of 0.25*σ*, 0.5*σ*, 0.75*σ*, *σ* and 1.25*σ*, and we simulated a 20% disease liability (--simu-cc 2100 8400 --simu-hsq 0.5 --simu-k 0.2). As a control, we also simulated a sixth locus with 5–10% MAF and effect size of 0. We had 100 iterations in total.

Genotypes at ascertained markers for subsets of cases and controls were selected using PLINK, and case/control associations were run in GEMMA using a MAF filter of 5% and a kinship matrix as a random effect. Within-breed GWAS designs were done using 100 (or 200) cases and controls from within a single breed, and two breeds were used for each of the 100 iterations. We used four different across-breed GWAS designs: balanced, random, semibalanced and unbalanced. For each of these, we used 100, 200, 500, and 1,000 cases and controls. A balanced GWAS had an equal number of cases and controls from each breed. In a random GWAS design, the cases and controls were chosen randomly across any of the 30 breeds using the linux command shuf. A semibalanced design had the number of cases from each of 20 breeds proportional to the prevalence in that breed. In an unbalanced GWAS, we used an equal number of cases and controls from each of 20 breeds, but this number was proportional to the prevalence in each breed.

The power of each GWAS was determined by counting the number of iterations (out of 100) that had detected the causal loci (within 1 Mb for across-breed designs, and within 5 Mb for within-breed designs) with a *P*-value cutoff of ≤5 × 10^−7^ (current array and 10k array) and ≤1 × 10^−7^ (dense 2 k array). These cutoffs are approximately 5% Bonferroni cutoffs, and were therefore similar to the thresholds we set for our complex disease GWAS. False discovery rates were calculated as the number of false-positive loci over the total number of significant loci (false positives and true positives) for each GWAS design and array. Plots of results were generated in R and IPython notebook using Matplotlib library.

## Additional information

**Accession codes**: Genotype and phenotype data have been deposited in Dryad (datadryad.org, doi:10.5061/dryad.266k4).

**How to cite this article:** Hayward, J. J. *et al*. Complex disease and phenotype mapping in the domestic dog. *Nat. Commun.* 7:10460 doi: 10.1038/ncomms10460 (2016).

## Supplementary Material

Supplementary Information Supplementary Figures 1-7, Supplementary Tables 1-3, Supplementary Methods and Supplementary References

Supplementary Data 1 Numbers of breeds used in each disease GWAS.

Supplementary Data 2 Values used in average-breed GWAS for morphological phenotypes.

## Figures and Tables

**Table 1:** Complex disease across-breed GWAS (CHD and ED) and within-breed GWAS (idiopathic epilepsy, GC, lymphoma, MCT and PSVA) results reaching genome-wide significance or near significance (PSVA).

**Disease**	**Number cases/number controls**	**Number or name of breeds**	**Top marker(s) (chr: position)**	***P*****-value**	**Freq. (cases)**	**Freq. (controls)**	**Candidate gene**
CHD (Norberg angle)	921	69	28: 34,369,342	4.9 × 10^−8^	n/a	n/a	*CTBP2*
ED	113/633	82	26: 16,554,6311: 77,938,330	2.6 × 10^−7^4.4 × 10^−7^	0.3580.367	0.1450.134	*POP5TLE1*
Idiopathic epilepsy	34/168	Irish Wolfhounds	4: 7.5–21	2.0 × 10^−8^	0.382	0.110	Many, including *ANK3*
GC	46/91	Boxers, French Bulldogs, American Bulldogs	38: 21.39–21.73	8.1 × 10^−10^	0.065	0.456	*SLAM* family members, *CD48*
Lymphoma	34/48	Golden Retrievers	4: 35,564,350	4.0 × 10^−7^	0.279	0.646	*MCC, MXD3, FGFR4*
MCT	152/106	Labrador Retrievers	36: 16,889,272	1.7 × 10^−7^	0.740	0.509	*ITGA6*
PSVA	57/101	Yorkshire Terriers	32: 14,626,183	1.2 × 10^−6^	0.675	0.361	
