# Supplementary material for: Complex disease and phenotype mapping in the domestic dog
Source: Nat Commun. 2016 Jan 22;7:10460. doi: 10.1038/ncomms10460 (PMC4735900; doi:10.1038/ncomms10460)
Supplement: Supplementary Information — Supplementary Figures 1-7, Supplementary Tables 1-3, Supplementary Methods and Supplementary References [file ncomms10460-s1.pdf]

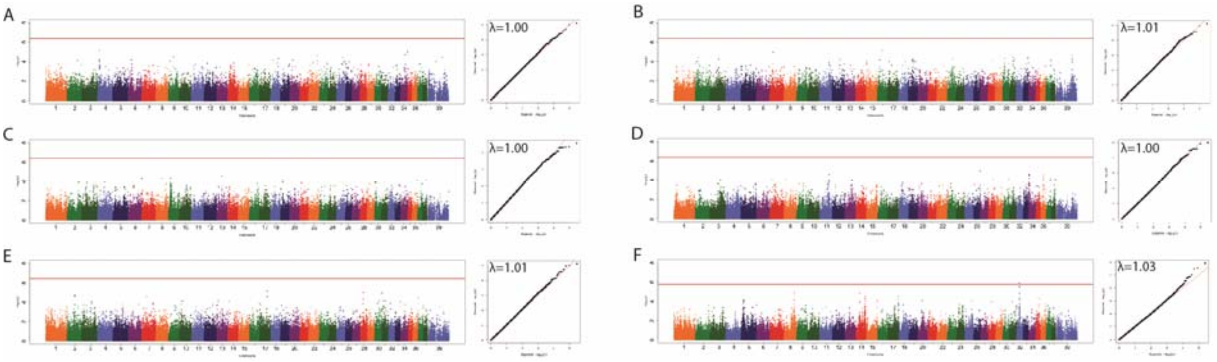

Supplementary Figure 1: Non-significant disease GWAS results.

Manhattan and quantile-quantile plots (with inflation factors, λ) for across-breed disease phenotypes A) CCLD B) lymphoma C) PSVA D) MCT E) MVD, where red lines are Bonferroni correction at $P = 4 \times 10^{-7}$, and plots for within-breed disease phenotypes F) PSVA in Yorkshire Terriers, where red line is 5% Bonferroni correction of unlinked SNPs.

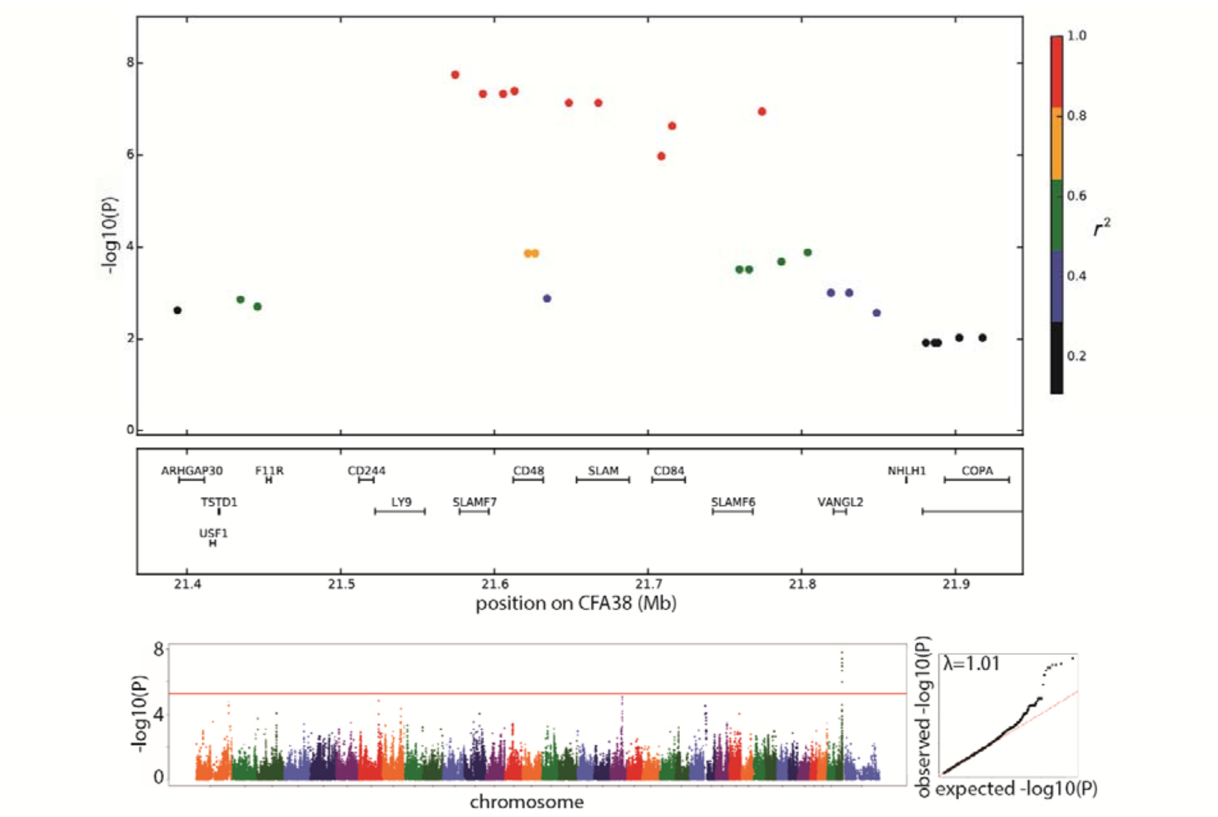

Supplementary Figure 2: GWAS of colitis in Boxers.

Manhattan and quantile-quantile plot of the colitis GWAS result in Boxers only. Colors of circles indicate amount of LD with most significantly associated SNP, ranging from black (r^2 = 0-0.2) to red (r^2 = 0.8-1). Red line on the Manhattan plot is 5% Bonferroni correction of unlinked SNPs. Inflation factor (λ value) is shown on the quantile-quantile plot.

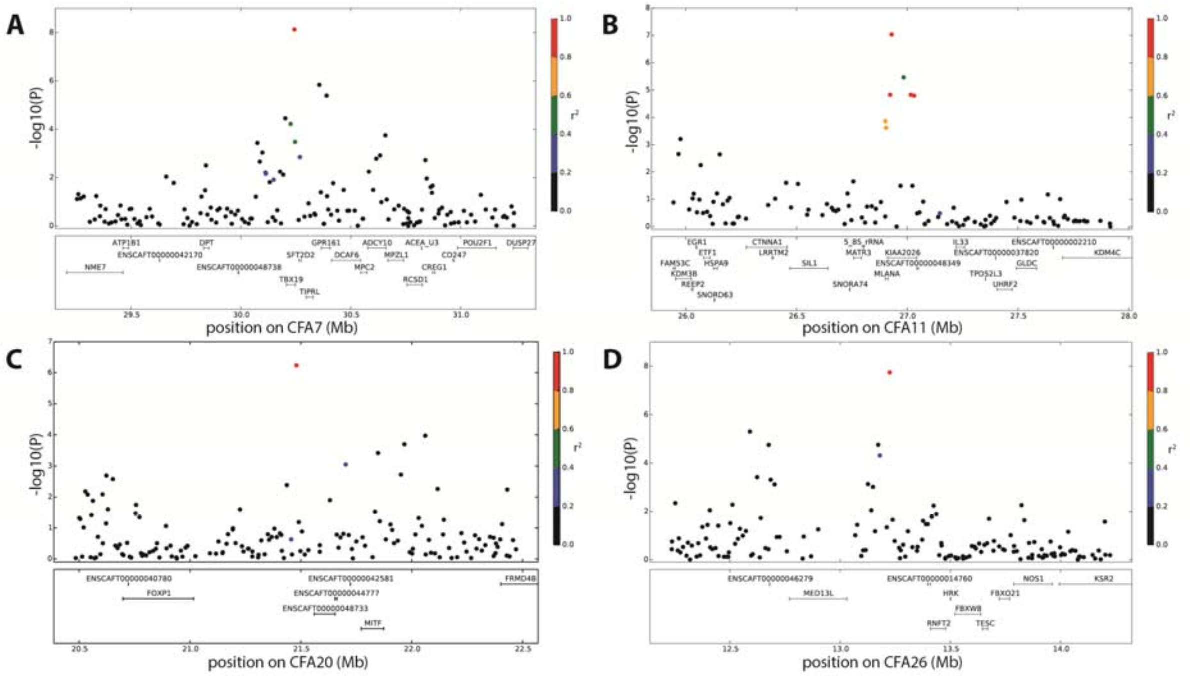

Supplementary Figure 3: LD plots for novel body size loci identified in breed-average weight GWAS.

A) CFA7 B) CFA11 C) CFA20 D) CFA26. Colors of circles indicate amount of LD with most significantly associated SNP, ranging from black (r^2 = 0-0.2) to red (r^2 = 0.8-1).

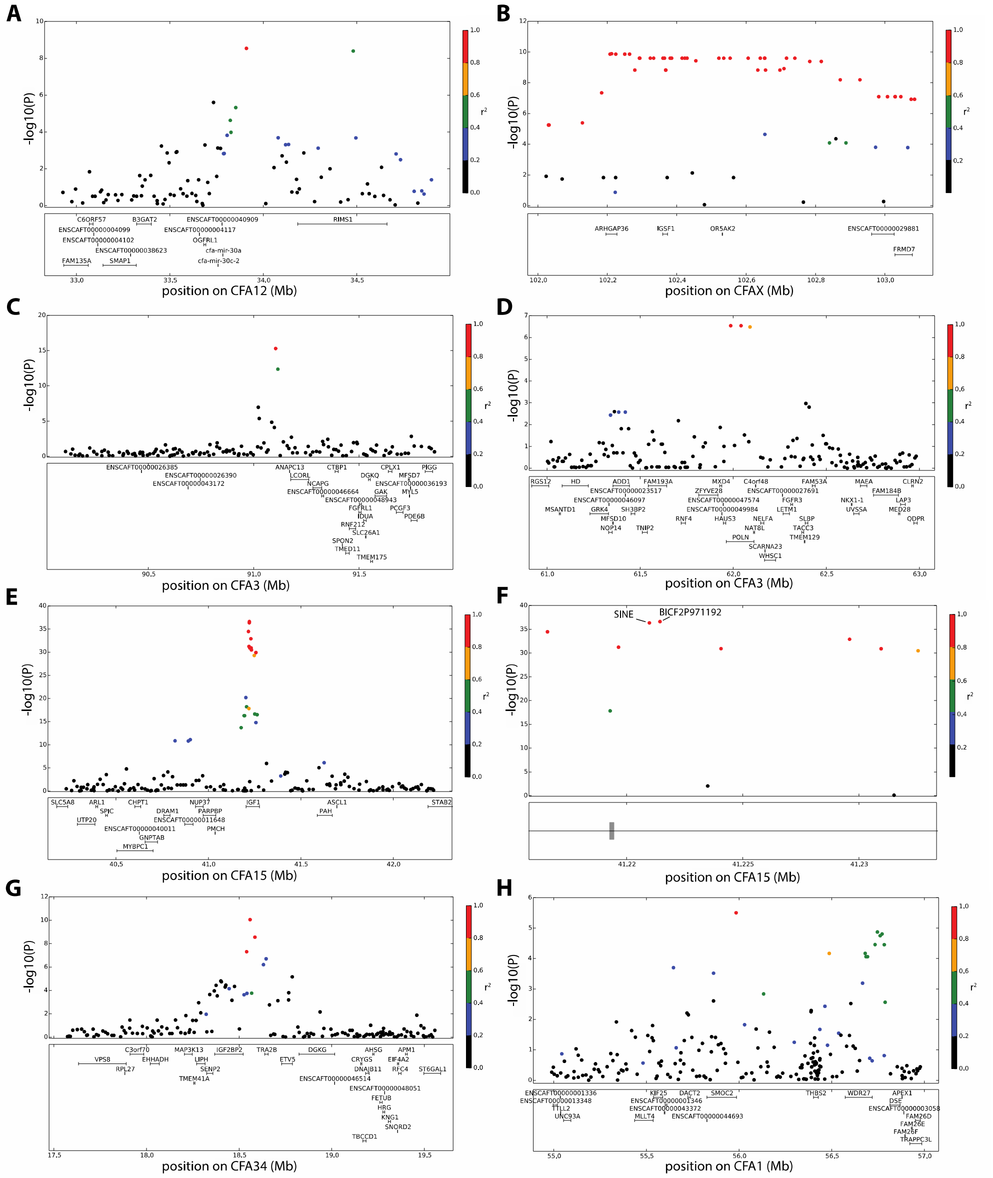

Supplementary Figure 4: LD plots for non-novel size loci identified in breed-average GWAS.

A) CFA12 (height GWAS) B) *IGSF1* C) *LCORL* D) CFA3 E) *IGF1* F) *IGF1* (box = exon) showing the locations of the directly-typed SINE and the “SNP 5” (BICF2P971192) variant in LD with the SINE [1] G) *IGF2BP2* H) CFA1 (*THBS2*/*SMOC2*). Colors of circles indicate amount of LD with most significantly associated SNP, ranging from black (r^2 = 0-0.2) to red (r^2 = 0.8-1).

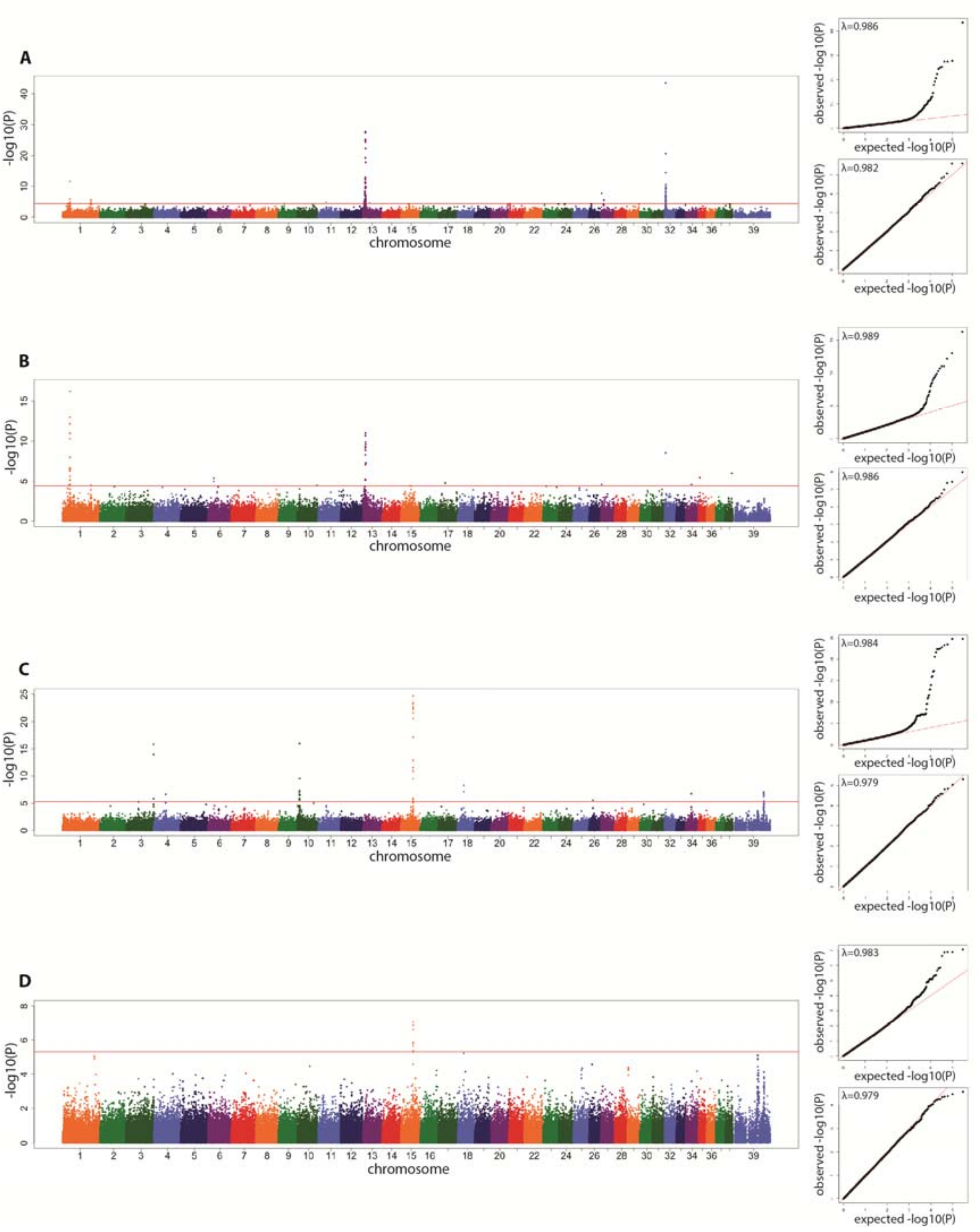

Supplementary Figure 5: Fur and Individual Weight Manhattan plots.

Breed-average phenotypes A) fur length B) shedding, and individual sex-corrected weight$^{0.38}$ for C) all dogs (n=2,072) D) village dogs (n=330). Two quantile-quantile plots are shown for each Manhattan plot: with all SNPs included (top) and with the significant SNPs removed (bottom) and inflation (λ) factors.

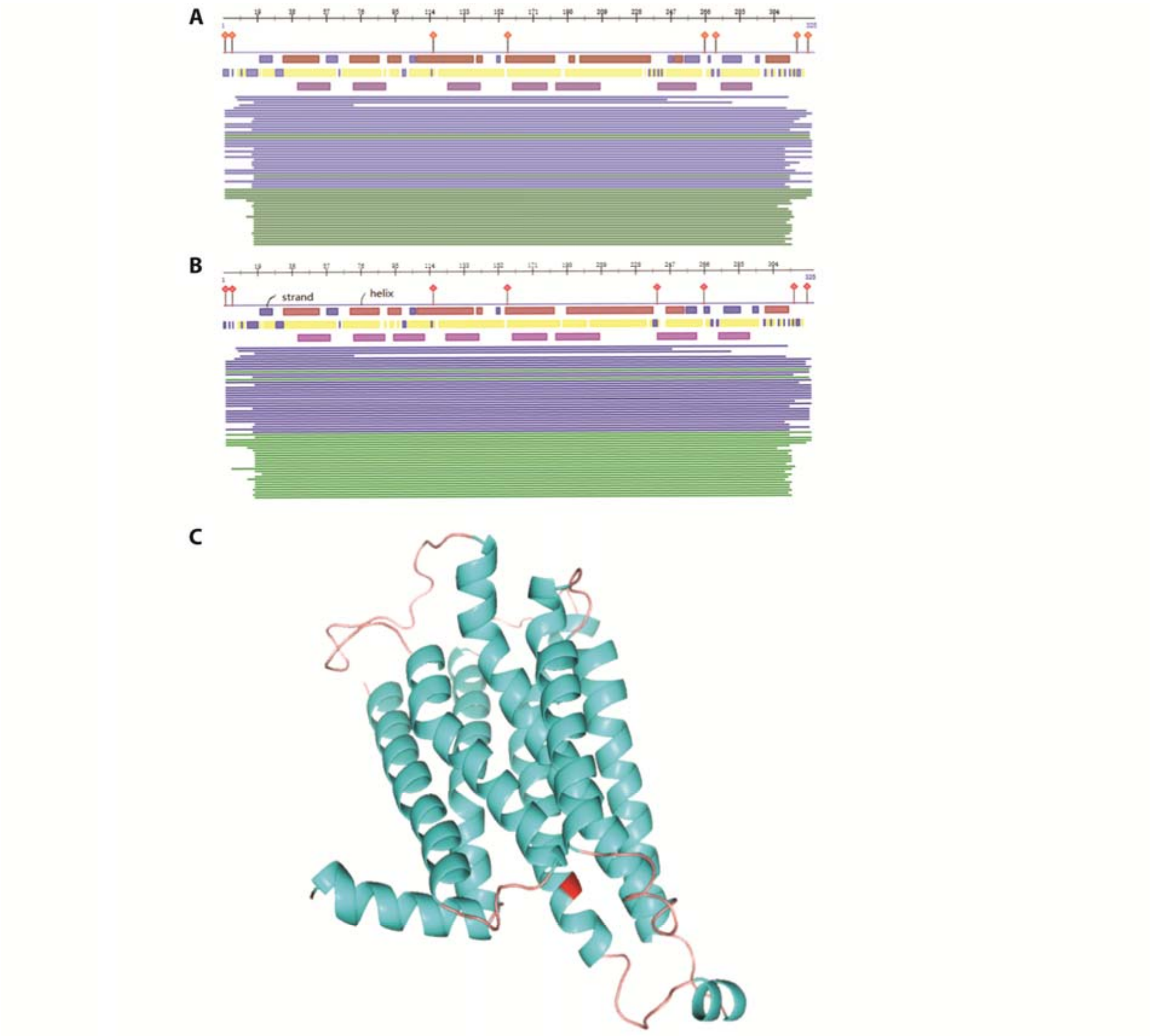

Supplementary Figure 6: MC5R protein.

Binding site prediction A) ancestral allele, and B) derived allele sequences. C) 3D structure of the MC5R protein, showing the missense mutation site in red.

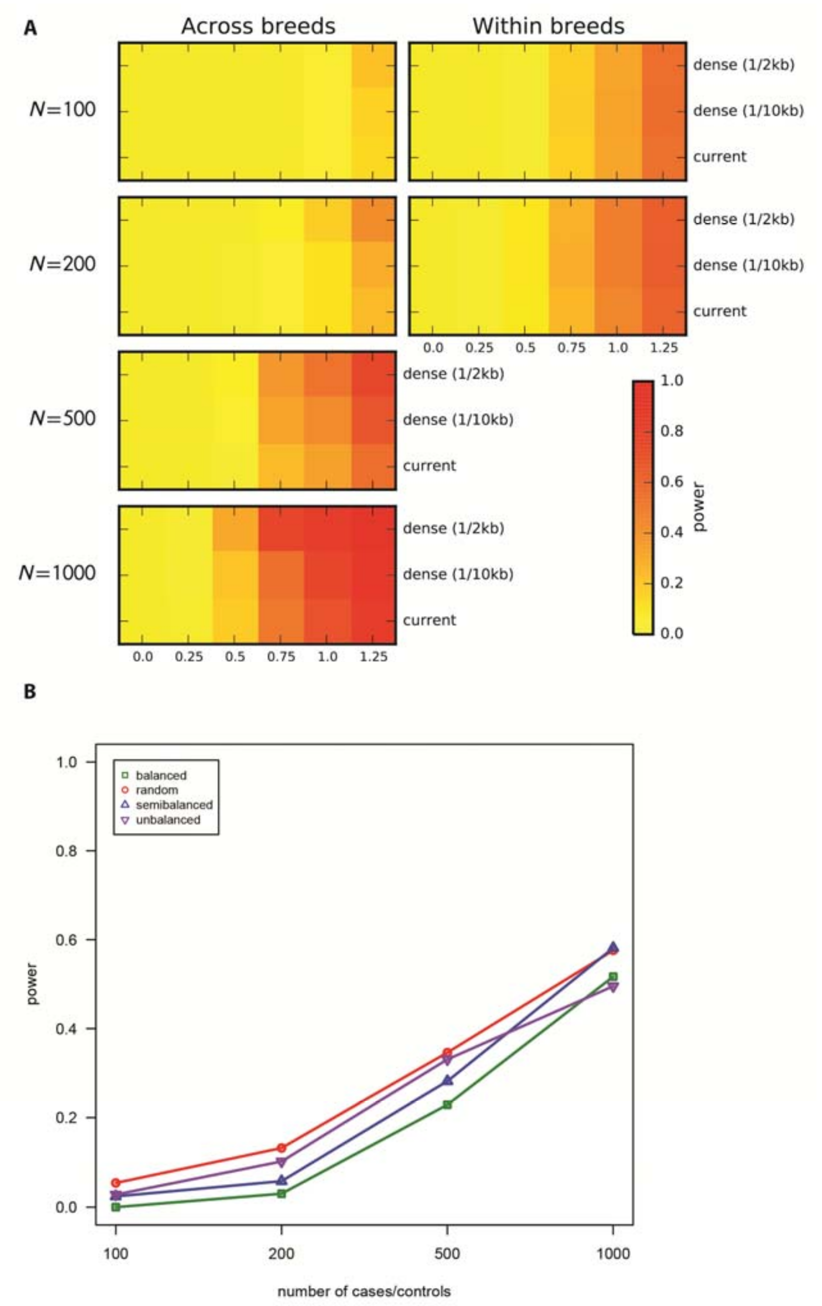

Supplementary Figure 7: Simulation GWAS results.

A) Power to detect causal loci of different effect sizes using a random across-breed design and within-breed design, different numbers of cases and controls, and different array densities. B) Power to detect causal loci using different GWAS across-breed designs (balanced, unbalanced, semi-balanced, random) and different numbers of cases and controls, with a dense (1 SNP every 2 kb) array. See methods for details.

Supplementary Table 1: Frequencies, effect size/odds ratio and *P*-values for SNPs associated with complex disease in different breeds.

Disease	Breed	N	SNP	Allele	Freq	Beta	SE	*P*-value
CHD	Golden Retriever	112	28:34,369,342	A	0.160	-6.580	1.87	$6.1x10^{-4}$
	Labrador Retriever	242			0.081	-6.500	1.44	$9.3x10^{-6}$
	German Shepherd Dog	82			0.006	3.346	10.58	0.753
	English Setter	79			0.000	n/a	n/a	n/a
	Newfoundland	59			0.085	-5.478	3.49	0.123
	Other	347			0.053	-3.593	0.02	0.022

Disease	Breed	N (cases/controls)	SNP	Allele	Freq (cases)	Freq (controls)	*P*-value	Odds ratio
ED	Labrador Retriever	30 / 180	1:77,938,330	G	0.917	0.972	0.032	0.314
	Golden Retriever	8 / 49			1.000	1.000	n/a	n/a
	German Shepherd Dog	10 / 45			0.500	0.744	0.031	0.343
	English Setter	27 / 52			0.000	0.087	0.026	0.000
	Other	38 / 307			0.816	0.933	0.0004	0.317
ED	Labrador Retriever	30 / 180	26:16,554,631	T	0.983	0.989	0.713	0.663
	Golden Retriever	8 / 49			0.688	0.918	0.007	0.196
	German Shepherd Dog	10 / 45			1.000	0.978	0.501	n/a
	English Setter	27 / 52			0.130	0.375	0.001	0.248
	Other	38 / 307			0.632	0.831	$3.16x10^{-5}$	0.350
lymphoma	**Golden Retriever**	34 / 48	4:35,564,350	A	0.279	0.646	$3.77x10^{-6}$	0.213
	Labrador Retriever	22 / 62			0.182	0.161	0.754	1.156
	Boxer	13 / 6			0.423	0.500	0.658	0.733
	Other	121 / 22			0.165	0.159	0.919	1.047
MCT	**Labrador Retriever**	152 / 106	36:16,889,272	G	0.740	0.509	$7.08x10^{-8}$	2.743
	Golden Retriever	31 / 9			0.710	0.778	0.569	0.698
	Vizsla	51 / 26			0.657	0.615	0.611	1.196
	Other	117 / 4			0.761	0.625	0.380	1.907
PSVA	**Yorkshire Terrier**	57 / 101	32:14,626,183	A	0.675	0.361	$7.93x10^{-8}$	3.678
	Maltese	26 / 24			0.731	0.854	0.130	0.463
	Norfolk Terrier	10 / 10			0.150	0.350	0.144	0.328
	Miniature Schnauzer	21 / 17			0.905	0.853	0.487	1.638
	Cairn Terrier	21 / 23			0.786	0.609	0.072	2.357
	Havanese	17 / 15			0.853	0.967	0.119	0.200
	Tibetan Spaniel	10 / 13			0.450	0.615	0.264	0.511
	Papillon	9 / 9			0.889	0.833	0.630	1.600

SE = standard error

Supplementary Table 2: GWAS results for breed average weight, individual weight, and breed average height.

Locus	Gene	Effect size ($kg^{0.38}$)	Weight breed avg original/covariate	Weight ind original/covariate	Height breed avg original/covariate	Reference
1:56.0-56.7	*THBS2/ SMOC2*	0.08	$3.4x10^{-6}$ / $1.1x10^{-4}$	$2.6x10^{-4}$ / $2.7x10^{-3}$	$2.8x10^{-9}$ / $6.4x10^{-8}$	2
3:41.7	*IGF1R*	0.07	$3.9x10^{-5}$ / $2.4x10^{-4}$	$6.9x10^{-6}$ / $2.8x10^{-6}$	$5.0x10^{-6}$ / $5.5x10^{-6}$	3
3:62.0		0.09	$3.2x10^{-7}$ / $1.5x10^{-5}$	$3.6x10^{-4}$ / $6.5x10^{-3}$	$2.2x10^{-7}$ / $1.6x10^{-7}$	4
3:91	*LCORL*	0.11	$5.8x10^{-16}$ / $7.5x10^{-14}$	$7.6x10^{-17}$ / $5.6x10^{-16}$	$2.5x10^{-10}$ / $4.5x10^{-9}$	5
4:39.1-39.4	*STC2*	0.08	$3.6x10^{-12}$ / $7.0x10^{-12}$	$7.2x10^{-8}$ / $1.4x10^{-6}$	$5.4x10^{-16}$ / $1.4x10^{-11}$	2,6
4:67.0[a]	*GHR*	0.04	$9.6x10^{-7}$ / $2.8x10^{-3}$	$5.0x10^{-3}$ / $1.1x10^{-2}$	$1.4x10^{-6}$ / $1.2x10^{-4}$	6
**7:30.2**	*TBX19*	0.07	$7.7x10^{-9}$ / $2.7x10^{-7}$	$1.5x10^{-3}$ / $1.0x10^{-3}$	$3.9x10^{-8}$ / $1.0x10^{-5}$	n/a
7:43.7-43.8	*SMAD2*	0.07	$9.1x10^{-17}$ / $8.5x10^{-10}$	$3.9x10^{-5}$ / $1.1x10^{-3}$	$1.5x10^{-15}$ / $1.2x10^{-6}$	2,4, 6
10:8.2	*HMGA2*	0.16	$7.5x10^{-35}$ / $1.7x10^{-32}$	$8.1x10^{-18}$ / $2.2x10^{-15}$	$7.6x10^{-27}$ / $7.7x10^{-23}$	2,4-6
**11:26.9**		0.06	$1.0x10^{-7}$ / $2.7x10^{-6}$	$1.9x10^{-5}$ / $4.3x10^{-4}$	$5.7x10^{-7}$ / $6.5x10^{-5}$	n/a
12:33.7-34.3	*OGFRL1*	0.06	$2.0x10^{-5}$ / $1.9x10^{-4}$	$7.2x10^{-4}$ / $9.9x10^{-4}$	$2.8x10^{-9}$ / $6.9x10^{-10}$	7
15:41.2	*IGF1*	0.18	$3.2x10^{-37}$ / $3.2x10^{-37}$	$1.4x10^{-25}$ / $1.4x10^{-25}$	$2.1x10^{-36}$ / $2.1x10^{-36}$	1,2,5,8,9
18:20.3	fgf4	0.12	$3.2x10^{-11}$ / $3.5x10^{-9}$	$4.2x10^{-9}$ / $7.4x10^{-7}$	$3.6x10^{-27}$ / $5.3x10^{-28}$	10
**20:21.5-22.0**	*MITF*	0.11	$6.0x10^{-7}$ / $5.6x10^{-7}$	$2.0x10^{-4}$ / $7.8x10^{-4}$	$7.2x10^{-6}$ / $1.6x10^{-5}$	n/a
**26:13.2[a]**		0.05	$1.9x10^{-8}$ / $1.8x10^{-4}$	$1.0x10^{-5}$ / $9.6x10^{-4}$	$2.4x10^{-7}$ / $5.6x10^{-5}$	n/a
34:18.6	*IGF2BP2*	0.08	$9.2x10^{-11}$ / $4.6x10^{-8}$	$4.7x10^{-8}$ / $2.5x10^{-4}$	$4.0x10^{-6}$ / $1.7x10^{-5}$	4
X:102.2-102.8	*IGSF1*	0.11	$1.5x10^{-10}$ / $7.4x10^{-9}$	$5.2x10^{-8}$ / $6.3x10^{-8}$	$1.2x10^{-5}$ / $7.0x10^{-5}$	2,5

Shown are the *P*-values for the original GWAS and the stepwise covariate GWAS. Effect sizes shown are for breed average weights in the stepwise covariate GWAS. Novel loci are listed in bold. Non-significant *P*-values are listed in grey.

[a] These loci are not significant in the stepwise covariate analysis.

Supplementary Table 3: Effect size and derived allele frequency of the 17 body size QTLs for breed dogs and village dogs.

Size QTL	Derived / ancestral alleles	Breed dogs: Effect size (weight/height) ($kg^{0.38}$/cm)	Breed dogs: Derived allele frequency	Village dogs: Effect size (weight/height) ($kg^{0.38}$/cm)	Village dogs: Derived allele frequency
1: 55983871	A / G	-0.13 / -1.59	0.102	0.07 / -0.04	0.060
3: 41758863	A / G	-0.01 / -0.42	0.342	-0.11 / -0.18	0.237
3: 61986452	A / G	0.24 / 2.79	0.121	0.21 / 0.68	0.099
3: 91103945	A / C	0.28 / 1.41	0.255	0.15 / 1.14	0.220
4: 39112085	C / G	-0.12 / -0.35	0.389	-0.01 / 0.05	0.289
4: 67026055[a]	A / G	-0.01 / 0.56	0.403	-0.11 / 0.23	0.411
**7: 30243851**	G / A	-0.07 / -0.50	0.243	-0.07 / 0.02	0.249
7: 43719549	G / A	-0.17 / -0.60	0.332	0.001 / -0.35	0.308
10: 8183593	A / G	-0.28 / -0.26	0.155	-0.06 / -0.72	0.175
**11: 26929946**	G / A	-0.10 / -0.74	0.240	-0.02 / -0.48	0.176
12: 33733595	G / A	0.07 / 0.29	0.483	0.12 / 0.95	0.434
15: 41229597	G / A	-0.36 / -2.95	0.419	-0.23 / -1.26	0.484
18: 20272961	C / A	-0.20 / -0.87	0.107	-0.27 / -2.34	0.122
**20: 21479863**	C / A	0.31 / 0.60	0.132	0.04 / 0.22	0.095
**26: 13224865[a]**	A / C	-0.07 / -1.42	0.325	-0.18 / -0.70	0.161
34: 18559537	A / G	-0.26 / -0.81	0.184	-0.10 / -0.59	0.143
X: 102212242	G / A	0.36 / 1.60	0.468	0.07 / -0.14	0.306

Effect size is calculated using an additive linear model of sex-corrected, inbreeding-corrected weights/heights. Novel loci are shown in bold.
[a] These loci are not significant in the stepwise covariate analysis.

Supplementary Methods: GENOME simulation parameters.

The GENOME program was run with the following command options:

```
-pop 32 100 100 1000 1000 1000 1000 1000 1000 1000 1000 1000 1000 1000 1000
1000 1000 1000 1000 1000 1000 1000 1000 1000 1000 1000 1000 1000 1000 1000
1000 1000 1000 -N population_history.txt -c 38 -pieces 25000 -len 2000 -s -1
-tree 0 -maf 0.001 -mut 1e-08 -rec 0.00001
```

With the following population_history.txt file:

```
0 15000 30000 2000 2000 2000 2000 2000 2000 2000 2000 2000 2000 2000 2000
2000 2000 2000 2000 2000 2000 2000 2000 2000 2000 2000 2000 2000 2000 2000
2000 2000 2000
1-1 2-2 3-3 4-4 5-5 6-6 7-7 8-8 9-9 10-10 11-11 12-12 13-13 14-14 15-15 16-16
17-17 18-18 19-19 20-20 21-21 22-22 23-23 24-24 25-25 26-26 27-27 28-28 29-29
30-30 31-31 32-32
4 15000 30000 250 290 330 370 410 450 490 530 570 610 650 690 730 770 810 850
890 930 970 1010 1050 1090 1130 1170 1210 1250 1290 1330 1400 1500
1-1 2-2 3-2 4-2 5-2 6-2 7-2 8-2 9-2 10-2 11-2 12-2 13-2 14-2 15-2 16-2 17-2
18-2 19-2 20-2 21-2 22-2 23-2 24-2 25-2 26-2 27-2 28-2 29-2 30-2 31-2 32-2
204 15000 30000
1-1 2-2
4204 15000 600
1-1 2-1
4354 15000
1-1
34354 15000
```

Supplementary References:

1. Sutter, N. B. *et al*. A single IGF1 allele is a major determinant of small size in dogs. *Science* **316**, 112-115 (2007).

2. Boyko, A. R. *et al*. A simple genetic architecture underlies morphological variation in dogs. *PLoS biology* **8**, e1000451 (2010).

3. Hoopes, B. C., Rimbault, M., Liebers, D., Ostrander, E. A. & Sutter, N. B. The insulin-like growth factor 1 receptor (IGF1R) contributes to reduced size in dogs. *Mammalian Genome* **23**, 780-790 (2012).

4. Jones, P. *et al*. Single-nucleotide-polymorphism-based association mapping of dog stereotypes. *Genetics* **179**, 1033-1044 (2008).

5. Vaysse, A. *et al*. Identification of genomic regions associated with phenotypic variation between dog breeds using selection mapping. *PLoS genetics* **7**, e1002316 (2011).

6. Rimbault, M. *et al*. Derived variants at six genes explain nearly half of size reduction in dog breeds. *Genome Res.* **23**, 1985-1995 (2013).

7. Quignon, P. *et al*. Fine mapping a locus controlling leg morphology in the domestic dog. *Cold Spring Harb. Symp. Quant. Biol.* **74**, 327-333 (2009).

8. Chase, K. *et al*. Genetic basis for systems of skeletal quantitative traits: principal component analysis of the canid skeleton. *Proc. Natl. Acad. Sci. U. S. A.* **99**, 9930-9935 (2002).

9. Eigenmann, J. E., Patterson, D. F. & Froesch, E. R. Body size parallels insulin-like growth factor I levels but not growth hormone secretory capacity. *Acta Endocrinol. (Copenh)* **106**, 448-453 (1984).

10. Parker, H. G. *et al*. An expressed fgf4 retrogene is associated with breed-defining chondrodysplasia in domestic dogs. *Science* **325**, 995-998 (2009).
